# PbS Colloidal Quantum Dot Photodetectors operating in the near infrared

**DOI:** 10.1038/srep37913

**Published:** 2016-11-25

**Authors:** Andrea De Iacovo, Carlo Venettacci, Lorenzo Colace, Leonardo Scopa, Sabrina Foglia

**Affiliations:** 1 NOOEL-Nonlinear Optics and OptoElectronics Lab, Dept. of Engineering, University Roma Tre, 00146, Rome, Italy; 2 CNR, Istituto dei Materiali per l’Elettronica ed il Magnetismo, Rome, Italy

## Abstract

Colloidal quantum dots have recently attracted lot of interest in the fabrication of optoelectronic devices due to their unique optical properties and their simple and low cost fabrication. PbS nanocrystals emerged as the most advanced colloidal material for near infrared photodetectors. In this work we report on the fabrication and characterization of PbS colloidal quantum dot photoconductors. In order to make devices suitable for the monolithic integration with silicon electronics, we propose a simple and low cost process for the fabrication of photodetectors and investigate their operation at very low voltage bias. Our photoconductors feature high responsivity and detectivity at 1.3 μm and 1 V bias with maximum values of 30 A/W and 2·10^10^ cmHz^1/2^W^−1^, respectively. Detectivity close to 10^11^ cmHz^1/2^W^−1^ has been obtained resorting to bridge sensor readout.

The near infrared (NIR) spectral range encompasses a wide variety of applications, including optical fiber communications, spectroscopy, imaging, security, remote sensing and metrology in several fields such as food inspection, agriculture, pharmaceutical and biology^1^.

Commercially available NIR detectors are based on epitaxial III-V semiconductor compounds such as InGaAs and InGaAsP. Their high material and technology cost per unit area prevents large scale deployment, but this can be overcome exploiting the combination of the optoelectronic functionality of III–V technology with the signal processing capabilities and advanced low-cost volume production techniques of silicon^2^. Unfortunately, material issues related to lattice mismatch, high thermal budget and cross contamination still hinder monolithic integration of III-V devices on silicon electronics^3^. Therefore, hybrid integration of InGaAs detectors with amplification and readout circuitry is preferred, increasing production costs and complexity^4^. The advantages of monolithic integration with silicon in terms of performance, low parasitics, reliability and compactness have been exploited with the development of CMOS compatible Ge on Si photodetector technology^5^,^6^. However, both the fabrication cost and the impact on the standard CMOS fabrication process are relevant and the operating spectral range, limited to less than 1600 nm (Ge direct bandgap), makes Ge/Si unsuitable for several applications that require longer wavelengths^7^.

The relatively new approach based on colloidal quantum dots (CQD) offers a very promising alternative under several aspects.

CQDs are semiconductor nanoparticles suspended in the solution phase that can be easily deposited by simple techniques. Thanks to their typical strong confinement, they exhibit enhanced light-matter interactions providing unique optical properties such as increased optical absorption and emission as well as size tunability^8^. For these reasons they have attracted a lot of attention for several optoelectronic devices, including light emitting diodes^9^, lasers^10^, solar cells^11^ and photodetectors^12^.

In addition, CQD, thanks to their solution processability, allow low cost, low temperature and large area fabrication, accepting a wide variety of substrates, including silicon, thus enabling their integration with electronics^13^.

Lead sulphide (PbS) quantum dots are the most advanced colloidal material for the NIR in terms of both monodispersion and reproducibility and CQD nanoparticles of several different size (with their corresponding spectral characteristics) are commercially available.

After the first demonstration of photosensitive devices based on CQD (photoconductivity of CdSe^14^ and PbS heterojunction photodiode^15^), in the past decade, lot of photodetectors have been proposed and demonstrated resorting to photodiodes, photoconductors and phototransistors. An updated and comprehensive review can be found in^16^.

Different device schemes have been proposed in order to meet the requirements of different applications. Photodiodes exhibit high speed and very low dark current. However, their sensitivity is limited by the fundamental limitation of <1 quantum efficiency^17^. Photoconductors exhibit very large responsivity thanks to their photoconductive gain but, on the other hand, show extremely low cutoff frequency and large dark current^18^. An alternative approach is based on phototransistors, a three terminal device where the gate potential is optically controlled, modulating the channel conductivity. The phototransistor combines the transistor gain with the low dark current while preserving high speed, at the cost of a more complex device architecture^19^.

The goal of our research is the development of NIR PbS CQD photodetectors suitable for the monolithic integration with silicon CMOS electronics with minimal or no impact on the fabrication process. We therefore resort to the photoconductor as the simplest planar device architecture that can be back-end integrated on electronic integrated circuits. In addition, in order to preserve the compatibility with the voltage levels typical of modern CMOS electronics, we focused on device operation in the low voltage bias regime (1 V).

After the impressive results of Konstantatos *et al*.^20^ (1.3 μm responsivity greater than 10^3^ A/W and detectivity exceeding 10^13 ^cmHz^1/2^W^−1^), several photoconductive detectors were proposed looking for the improvement of the device performance and a suitable balance between sensitivity and speed^16^.

Several studies have been conducted aiming to the control of material doping, carrier mobility and lifetime often exploiting different ligands and engineering oxidation processes of the QDs^18^,^21^,^22^,^23^,^24^. Such research works lead to significant advance in the field and relevant device performance were demonstrated. However, the reported results are hard to compare since in CQD photoconductors different parameters such as sensitivity and speed cannot be optimized simultaneously and one can be maximized at the expense of the other, according to specific application needs. In addition, several device characteristics depend on voltage bias, wavelength, optical input power and device area. With the exception of very few cases^25^,^26^, high performance has been obtained at very high voltage bias (10 to 100 V) that are unpractical for several applications and are not compatible with silicon integrated circuits. Moreover, most papers do not report responsivity in the near infrared and report on devices characterized by means of visible light illumination.

In this work we focus on photoconductors fabricated from commercially available PbS CQDs following a very simple processing based on ligand exchange and drop casting the CQDs on interdigitated gold contacts deposited on oxidized silicon.

Device characterizations were performed in terms of current-voltage measurements in the low bias regime in dark and under optical excitation at 1.3 μm. The most relevant device parameters, such as responsivity and detectivity were measured at different voltage bias and different optical power. The time response is reported as well. We also investigated different device readout approaches for dark current cancellation and noise reduction, including resistive bridge configuration and impedance spectroscopy.

## Results and Discussion

**Figure 1:**
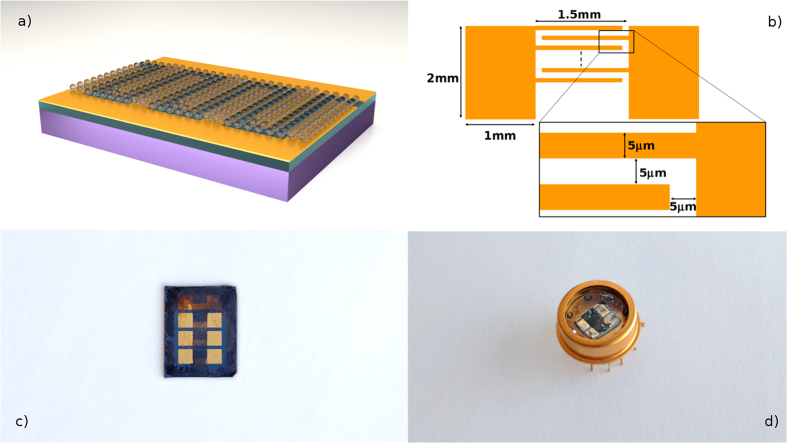
Device schematic and pictures. (**a**) Device artwork showing PbS nanoparticles on a couple of interdigitated Au contacts deposited on oxidized silicon. (**b**) Geometry and dimensions of the metal contacts. (**c**) Picture of an array of PbS CQD photoconductors. (**d**) Packaged devices.

In this section we report on the characterization of the PbS CQD photoconductors shown in Fig. 1, based on current-voltage measurements taken with a probe station based on a KEITHLEY source measure unit 2636B. Other measurement techniques will be described in the final part.

**Figure 2:**
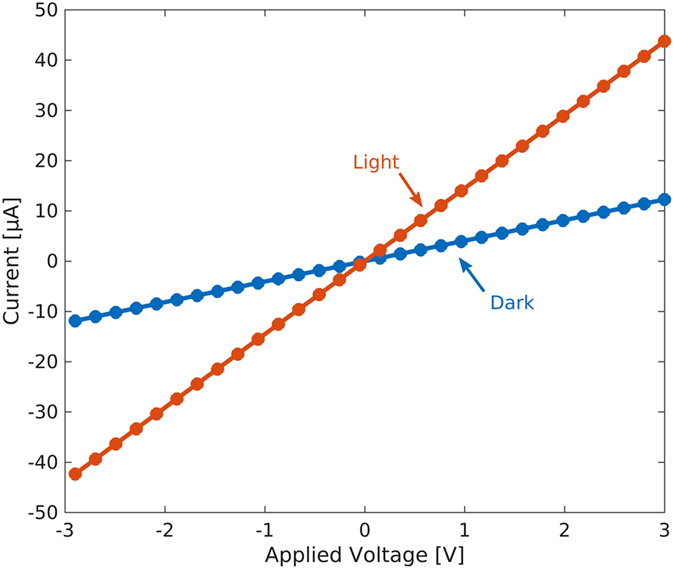
Current-voltage characteristics. Dark current (blue line) and total current (red line) under surface illumination at 1.3 μm and 10 μW optical power.

Typical current-voltage characteristics of our PbS CQD photoconductors are shown in Fig. 2 in dark condition and under illumination from a fiber coupled laser at 1.3 μm. Good ohmic contacts were observed in all samples, as demonstrated by the linear and symmetrical behavior of the dark current. This was expected since PbS CQD are known to be p-type, due to the presence of oxygen species that form shallow acceptor states, and therefore form ohmic contact with high work function metal such as Au^27^. The typical dark resistance *R*_*d*_ and dark current density *J*_*d*_ are about 250 kΩ and 0.3 mA/cm^2^, respectively, the latter being measured at 1 V. The measured conductivity confirms the effective removal of the original long chain organic ligands.

Figure 2 also shows the photocurrent when the device is surface illuminated at 1.3 μm and 10 μW optical power. The linear behavior with the applied voltage is in agreement with the classical photoconductive transport of a primary photocurrent originating by absorption, electron-hole generation and collection (limited to unity quantum efficiency) and a secondary photocurrent resulting from the injection and transit of carriers governed by the ratio between their transit time and lifetime that can produce photoconductive gain. Ligand exchanged PbS CQD are known to have midgap trap states that act as sensitizing recombination centers increasing the electron lifetime thus producing a field dependent gain^28^.

**Figure 3:**
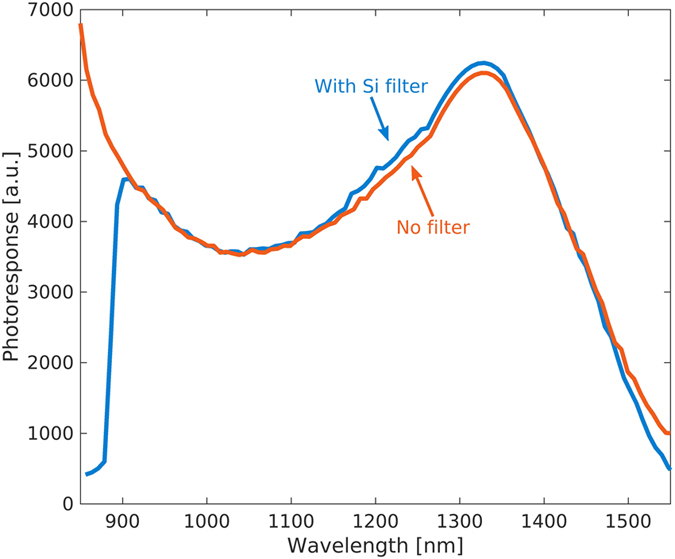
Photodetector spectral response. Typical spectral response measured by a scanning monochromator with a light intensity in the 50 μW/cm^2^ range and 1 V applied voltage (red line) and spectral response when a silicon filter is used to cover the device package (blue line).

The spectral response of the PbS photoconductors is reported in Fig. 3 (red line) as measured by a scanning monochromator with a light intensity in the 50 μW/cm^2^ range and 1 V applied voltage. The spectrum closely reproduces the absorption spectrum (not shown) and the excitonic peak at 1320 nm. A significant photoresponse up to 1.5 μm is observed. Figure 3 also shows the spectral response of the photodetector (blue line) when a silicon filter is used to cover the device package demonstrating that such configuration can be effectively employed for visible-blind NIR detection.

**Figure 4:**
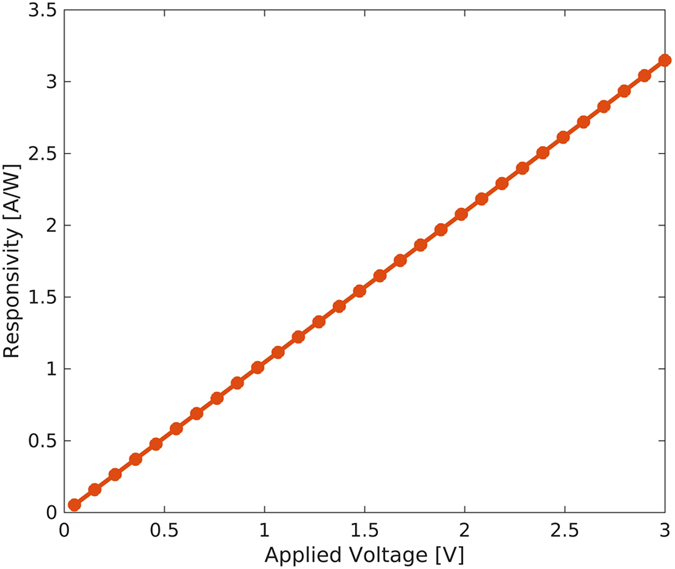
Responsivity versus bias voltage. Responsivity *R* evaluated from the net measured current reported in Fig. 2, according to the equation (2). Optical excitation is at 1.3 μm and 10 μW optical power.

**Figure 5:**
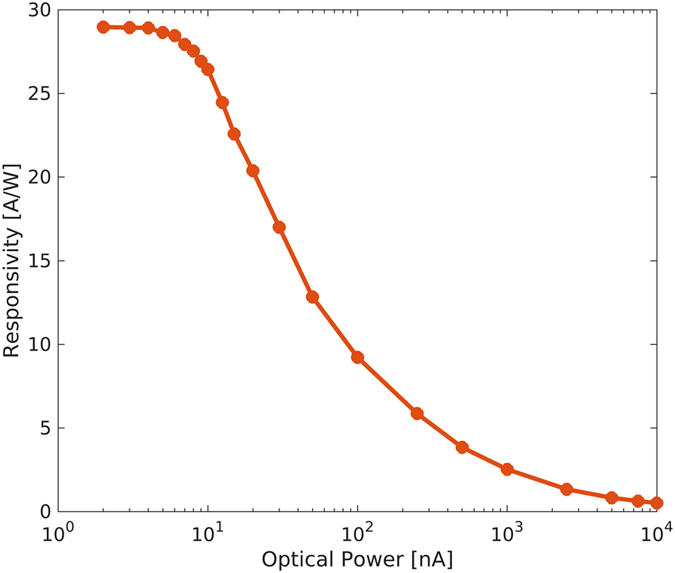
Responsivity versus optical power. Responsivity is measured at 1 V applied voltage in the 2 nW–10 μW range.

Figure 4 shows the detector responsivity *R* versus applied voltage as evaluated from the net measured current reported in Fig. 2, according to the following equation:


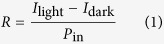


where *I*_*light*_ is the total current under illumination, *I*_*dark*_ is the dark current and *P*_*in*_ is the optical input power. Devices exhibit a typical responsivity of 1 A/W at 1.3 μm and 10 μW optical excitation and 1 V bias, but it should be considered that *R* is strongly dependent on both optical power and applied voltage. As mentioned in the introduction, in order to study the PbS CQD detectors in view of their potential integration with silicon CMOS integrated circuits, we now investigate the device performance at very low bias. Figure 5 shows the device responsivity versus the incident optical power measured at 1 V bias. The responsivity exhibits a strong power dependence reaching a maximum value of about 30 A/W at powers below 5 nW with values >10 A/W in the 1–100 nW range. Such performance is comparable with previously reported similar devices^20^ and compares favorably with most results reported at low applied voltage^25^,^26^. The large responsivity is associated to the large gain resulting from a long electron lifetime compared to transit time between the metal contacts. The strong dependence of the responsivity on the intensity of the incident light is typical of ligand exchanged PbS CQD photoconductors. The photoconductive gain is related to the effectiveness of the mid-gap states to capture electrons, which depends on their occupancy and therefore on light intensity. At low level illumination, the photogenerated carrier density is expected to be lower than the trap density thus producing higher gain. At increasing light intensity, the increasing trap filling raises the electron quasi Fermi level reducing the capture rate thus decreasing the gain.

A linear optical to electrical conversion is generally preferred, especially in metrology^29^. However, a saturated gain with a large responsivity at low optical power is interesting in several applications that require high sensitivity at very low signal level (such as sensing and imaging). The gain compression associated to the responsivity reduction at larger optical powers can be effectively employed when signal with large dynamic range must be sensed without the need for additional logarithmic amplifiers^30^.

The overall photodetector performance can be assessed referring to the sensitivity, namely the detector’s ultimate capability to detect small signals, i.e. the minimum optical power a photodetector can read from noise. Achieving high sensitivity requires both large responsivity and low noise. The most common figure of merit for sensitivity is the specific detectivity D* defined according to the following equation:


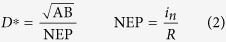


where *A* is the device area, *B* is the electrical bandwidth, *NEP* is the noise equivalent power given by the ratio between the noise current *i*_*n*_ and the responsivity *R*.

The noise current *i*_*n*_ of our PbS photoconductors has been measured resorting to RMS evaluation after verifying the series are stationary ergodic by taking measurements over different time spans. Typical RMS noise currents are in the nA range at 1 V bias.

**Figure 6:**
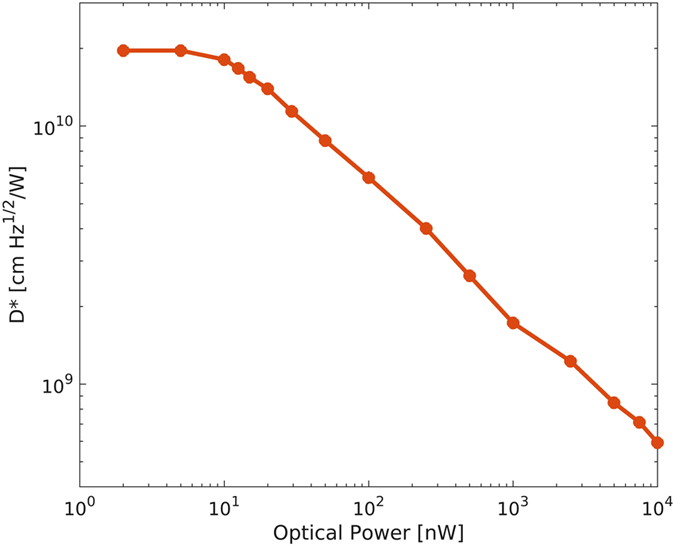
Specific detectivity D* versus optical power. Detectivity is measured at 1 V applied voltage in the 2 nW–10 μW range.

Figure 6 shows the typical specific detectivity as evaluated by equation (2), plotted versus the optical power when 1 V bias is applied. Best devices exhibit a detectivity as high as 2·10^10^ cmHz^1/2^W^−1^ at 1.3 μm and 1 V bias, for optical power <5 nW. Such value compares well with those reported in literature at low voltage bias^18^,^25^,^26^.

**Figure 7:**
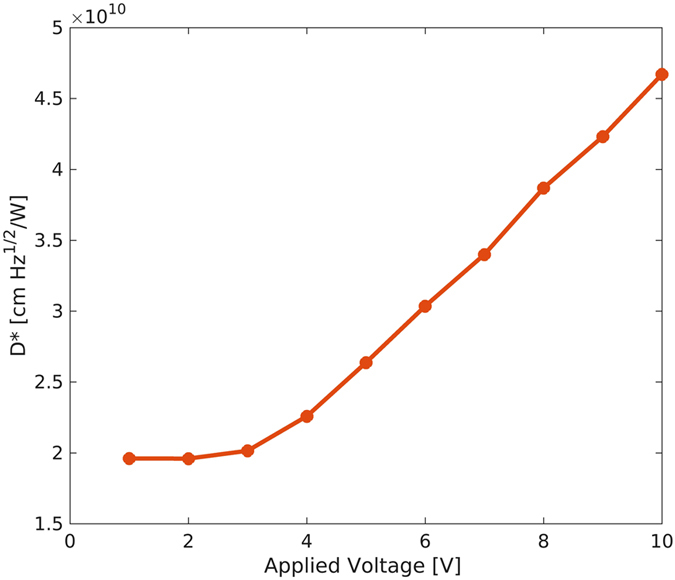
Specific detectivity D* versus voltage bias. Detectivity is measured at 5 nW optical power and applied voltage in the 1–10 V range.

We also investigated the role of the applied voltage on D*. A typical result is shown in Fig. 7 for 5 nW optical power. Detectivity is initially constant whereas it starts to linearly increase with the applied voltage above 2 V. A maximum detectivity of 4.5·10^10 ^cmHz^1/2^W^−1^ has been obtained at 10 V. Different D* behaviors with applied voltage (monotonic, non monotonic and no substantial change) have been reported in literature and the issue is still under investigation^20^,^31^. We observed that the noise current does not significantly depend on the on the optical power, suggesting the photocurrent shot noise contribution is negligible. In addition, the thermal noise current, as evaluated by 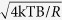 is negligible as well (≈1 pA). Therefore, the total noise should be dominated by the dark current shot noise and *1/f* excess noise (both related to the device dark current)^32^.

For this reason, significant efforts have been devoted to the reduction of the dark current in PbS CQD photoconductors, resorting to both blending with metal nanoparticles and using different ligands^16^. However, decreasing the dark current while preserving the photocurrent (or responsivity) proved to be very challenging since the two characteristics typically change unidirectionally. For example, by using silver nanocrystals, a dramatic decrease of the dark current has been obtained (nA range) at the cost of a low responsivity (<0.1 A/W)^23^. Sometimes, a more favorable balance between dark current and responsivity was achieved, leading to device with D* in the 10^12^–10^13 ^cmHz^1/2^W^−1^ range (although operated at relatively high voltage bias)^18^,^31^. Such approaches make the device fabrication more complex and may reflect on reduced repeatability and reliability. For this reason, in this study, we preferred to keep the fabrication process as simple as possible and try to reduce the dark current effects resorting to more sophisticated sensor readout approaches, namely the impedance measurement and the bridge configuration.

**Figure 8:**
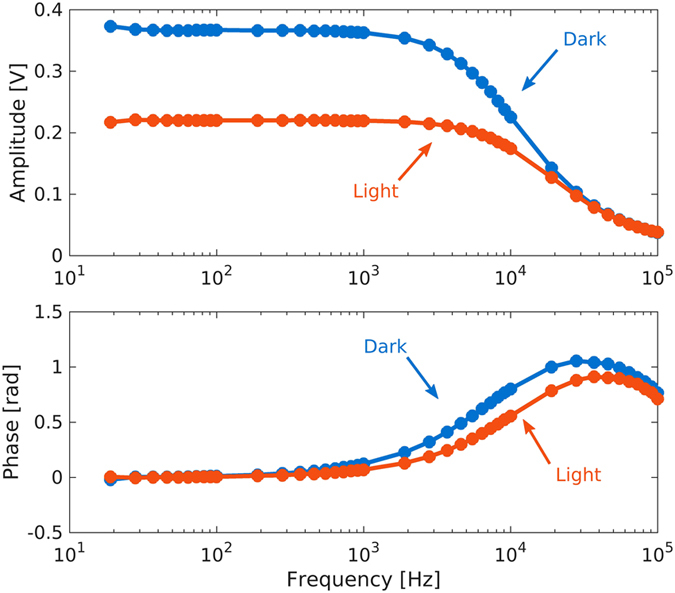
Response of PbS CQD photodetectors. Measurement are performed by a lock-in amplifier with a series resistor of 100 kΩ and a sinusoidal excitation. Amplitude and phase are reported versus frequency under both dark (blue line) and 10 μW illumination (red line).

The impedance measurement of the PbS CQD photodetectors was performed by a Stanford Research SR830 lock-in amplifier with a series resistor of 100 kΩ and using a sinusoidal signal excitation. Amplitude and phase of the measured impedance are reported in Fig. 8 versus frequency under both dark and 10 μW illumination. The measurement substantially yields the same result up to 1 kHz with most response associated to the amplitude. The device responsivity can be defined as the ratio between the net voltage (*V*_*light*_ − *V*_*dark*_) and the incident optical power. Typical responsivities above 10^5^ V/W have been measured at low optical power. Responsivity exhibited a linear increase with the amplitude of the measurement signal. In order to evaluate the detectivity achievable with this approach, we measured the voltage noise *v*_*n*_ in dark condition and we used equation (2), where *v*_*n*_ replaces *i*_*n*_ and the responsivity is expressed in V/W. Maximum detectivity of 6·10^10^ cmHz^1/2^W^−1^ has been obtained and we did not observe significant change with the applied voltage (in the low bias regime).

The bridge measurement is based on a Wheatstone configuration made of two photodetectors (one exposed to light while the other is blinded) and two adjustable resistors. The bridge is provided with a DC supply and the voltage is measured by the KEITHLEY source measure unit. The resistors are trimmed in order to set the voltage across the bridge to zero in dark condition and to set a voltage drop on the photodetector of 1 V. It is worth noting that such dark signal cancellation is very effective in terms of drift over long time due to the presence of the blind detector. The voltage across the bridge is finally measured under illumination. Since the measured signal is a voltage, responsivity and detectivity are defined in the same way as the lock-in measurement. Maximum *R* and D* are 2·10^6^ A/W and 10^11^ cmHz^1/2^W^−1^, respectively.

**Figure 9:**
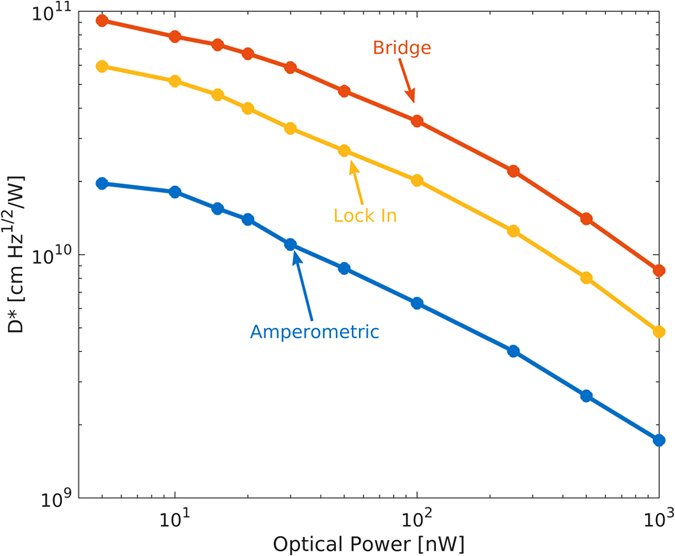
Comparison of D* obtained by different device readout techniques. Three different measurement approaches (current, impedance and bridge) are compared in terms of the detectivity versus optical power with 1 V applied voltage.

The three different measurement approaches (current, impedance and bridge) are compared in Fig. 9 in terms of the achieved detectivity versus optical power with 1 V applied voltage. The bridge measurement allows a fivefold improvement over the basic current measurement whereas the lock-in provides a factor of three. Better results were expected from the impedance measurement thanks to the lower noise provided by the small bandwidth measurement of the lock-in. However, we noticed that the responsivity is relatively smaller than in the other cases (where the device is DC biased).

Our PbS photoconductors exhibit relatively high detectivity at low voltage bias but, in photodetection, the time response is important as well. The large responsivity depends on the photoconductive gain achieved by the large carrier lifetime associated to the trap states. Unfortunately, the time response of photoconductors is dominated by recombination times and large lifetime severely limits the device speed. Therefore a suitable tradeoff between sensitivity and speed must be found. The energy levels associated with the trap states responsible for large gain have been investigated and three centers at 0.1, 0.2 and 0.3 eV below the conduction band were found, corresponding to 60, 300 and 2000 ms time constants, respectively^28^,^33^. Several approaches have been proposed for the improvement of the time response, including the selective introduction of surface trap states^33^ or PbS encapsulation with inorganic compounds^18^. However, a suitable balance between gain and time response is still quite challenging.

**Figure 10:**
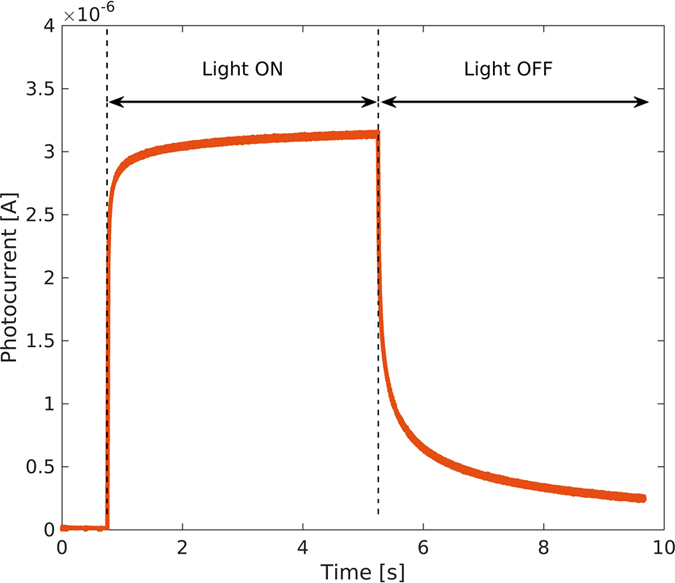
Typical photodetector time response. The device speed is measured by a pulsed laser at 1.3 μm wavelength and 1 μW optical power, biasing the photodetectors at 1 V.

In this study, in order to keep the device fabrication as simple as possible, we did not resort to any additional chemical processing. The device speed was measured by a pulsed laser at 1.3 μm wavelength and 1 μW optical power, biasing the photodetectors at 1 V. The typical time response is reported in Fig. 10. Using a multiple exponential fit, we extracted the following time constants: 30 ms, 300 ms and 2800 ms that are in good agreement with those previously reported on similar devices by other authors^28^. The rise and fall times, according to the traditional definition as the time taken by the signal to change from 10% to 90% of the final value, are 160 ms and 3 s, respectively, the latter accounting for a device bandwidth of about 0.1 Hz. Such time response is unsuitable for some applications but can be compatible with several sensor applications, namely chemical and gas sensors, where a tradeoff between sensitivity and speed largely moved to the former can be accepted.

## Conclusions

In summary, we have fabricated PbS CQD photoconductors by a simple preparation and deposition technique based on ligand exchange and drop cast of the quantum dots on coplanar interdigitated contacts on oxidized silicon chip. We investigated the device performance in terms of responsivity, detectivity and time response at voltage bias as low as 1 V. The PbS photoconductors exhibit high responsivity and detectivity at 1.3 μm with maximum values of 30 A/W and 2·10^10 ^cmHz^1/2^W^−1^, respectively.

Other sensor readout approaches, including a bridge configuration and an impedentiometric measurement, were attempted and detectivity was increased up to 10^11^ cmHz^1/2^W^−1^. The device time response was quite slow, with rise and fall times of 160 ms and 3 s, respectively.

In conclusion, the overall performance obtained at low voltage bias, combined with the simple fabrication process on silicon, make the proposed device suitable for the realization of NIR photodetectors integrated on silicon integrated circuits.

## Methods

Photodetectors have been fabricated by drop casting a CQD solution directly onto interdigitated gold contacts. This approach ensures a very low material waste and completely eliminates the need to deposit and pattern metal over the QD film. PbS is, in fact, extremely sensitive to the oxidizing and etching action of aqueous solutions normally employed for lithographic patterning, thus we preferred to deposit the photosensitive film as the last step of the device fabrication process. Compared to other deposition methods such as spin coating or Langmuir-Blodgett auto-assembly, drop casting the CQD solution directly on metal contacts ensures a higher spatial selectivity and superior scalability; moreover, the deposition system can be engineered to fit into an inkjet printing device. Such approach could lead to the realization of a multi-material deposition process aiming at the fabrication of different photodetectors at the same time, each one with a different optical response.

For the device fabrication process, we employed a commercial 10 mg/ml solution of PbS quantum dots capped with oleic acid and dispersed in toluene. The first excitonic absorption peak of the employed QDs was in the near infrared at 1320 nm. In order to decap the nanoparticles of the organic ligands, methanol was added to the colloid in nitrogen atmosphere. Particle precipitation was obtained centrifuging the solution at 10000 rpm for 8 minutes; methanol addition and centrifugation were repeated twice in order to ensure complete removal of the long chain oleic acid ligand. After centrifugation, the sample was dried in vacuum for 24 hours. Eventually, the nanoparticles were redispersed in octane with a concentration of 0.83 mg/mL. Even without any capping agent, the colloid proved to be stable for a few weeks if cooled and kept in inert atmosphere. The QD solution was drop-casted onto pre-patterned, 150 nm thick interdigitated Cr-Au contacts. The metal contacts were evaporated on thermally oxidized silicon wafers (t_ox_ = 1.5 μm) and patterned by standard optical lithography. The fingers are 1.5 mm long with 5 μm width and spacing, accounting for a device area of 1.5 mm^2^. Devices are provided with 2 mm^2^ bonding pads. The device schematic and geometry is reported in Fig. 1. The CQD deposition was performed in nitrogen atmosphere in order to avoid any oxidation of the nanoparticles. The colloid drop was dried in vacuum until full solvent evaporation; subsequently, a drop of butylammine was deposited onto the device and let react with the PbS until full evaporation (in vacuum). Butylamine reacts with PbS linking to the nanoparticles surface and redistributing them on the substrate; after evaporation, the resulting mean distance between neighboring particles should be almost twice as long as a butylamine molecule (~ 0.6 nm).

The deposition was repeated several times in order to obtain the desired film thickness (~150 nm). Finally, the devices were dipped in methanol for 2 hours in order to remove the butylamine and expose the surface of the nanocrystals. Methanol acts as a nonsolvent for PbS thus removing the organic ligand and further reducing the mean distance between nanoparticles. Moreover, once the PbS surface is exposed it can be oxidized in order to create the mid-gap, long-lived, trap states responsible for device sensitization and photoconductive gain enhancement^20^. The oxidation is obtained leaving the devices at room temperature in ambient air for 24 hours.

We employed CQD solutions with different particle concentration and we observed that reducing the colloid density dramatically improved the repeatability of the deposition process even if slightly hampering the photodetectors responsivity. We obtained a satisfactory tradeoff with a 0.83 mg/ml solution.

With respect to previously reported similar fabrication procedures, we exploited neither a liquid- nor solid-phase ligand exchange; conversely, we employed a hybrid technique first removing the long chain oleic acid in the liquid phase and then linking butylamine to PbS QDs directly on the deposition substrate. In this way, we keep the solution processing as simple and fast as possible but, at the same time, we limit the number of washing and soaking steps usually needed in solid-phase ligand exchange processes.

## Additional Information

**How to cite this article**: De Iacovo, A. *et al*. PbS Colloidal Quantum Dot Photodetectors operating in the near infrared. *Sci. Rep*. **6**, 37913; doi: 10.1038/srep37913 (2016).

**Publisher’s note:** Springer Nature remains neutral with regard to jurisdictional claims in published maps and institutional affiliations.
